# NT-proBNP for Predicting All-Cause Death and Heart Transplant in Children and Adults with Heart Failure

**DOI:** 10.1007/s00246-024-03489-7

**Published:** 2024-05-09

**Authors:** Walter Schmitt, Christian Diedrich, Taye H. Hamza, Michaela Meyer, Thomas Eissing, Stefanie Breitenstein, Joseph W. Rossano, Steven E. Lipshultz

**Affiliations:** 1 Pharmacometrics, Bayer AG, Wuppertal, Germany; 2 Pharmacometrics, Bayer AG, Leverkusen, Germany; 3 HealthCore, Watertown, MA USA; 4 Pediatric Medicine, Bayer AG, Wuppertal, Germany; 5 Division of Cardiology, Perelman School of Medicine, Children’s Hospital of Philadelphia, University of Pennsylvania, Philadelphia, PA USA; 6 Department of Pediatrics, Jacobs School of Medicine and Biomedical Sciences, Clinical and Translational Research Center, University at Buffalo, 875 Ellicott Street, Suite 5018, Buffalo, NY 14203 USA

**Keywords:** N-terminal pro hormone B-type natriuretic peptide, Heart failure, Cardiomyopathy, Cardiac death, Pediatric

## Abstract

Plasma N-terminal prohormone B-type natriuretic peptide (NT-proBNP) concentration is a heart failure (HF) biomarker in adults and children. Its prognostic value for HF-related events has been established only in adults. Therefore, we aimed to test the hypothesis that plasma NT-proBNP concentrations predicted the risk of heart transplantation or death in children with HF. We studied the medical records of 109 children with HF enrolled in the IBM Watson Explorys database and from 150 children enrolled in the Pediatric Cardiomyopathy Registry (PCMR). Nonlinear regression was used to assess the relationship between plasma NT-proBNP concentrations and the risk of events in the two cohorts. All children in the PCMR cohort had dilated cardiomyopathy. The Explorys cohort also included children with congenital cardiovascular malformations. Median plasma NT-proBNP concentrations were 1250 pg/mL and 184 pg/mL in the Explorys and PCMR cohorts, respectively. The percentage of deaths/heart transplantations was 7%/22%, over 2 years in the Explorys cohort and 3%/16% over 5 years in the PCMR cohort. Mean estimates of plasma NT-proBNP concentration indicative of half-maximum relative risk for events (EC_50_ values) at 2 and 5 years were 3730 pg/mL and 4199 pg/mL, respectively, values both close to the mean of 3880 pg/mL established for adults with HF. The plasma NT-proBNP concentration is suitable for estimating relative risk of mortality and heart transplantation in children with HF, independent of etiology and shows similar relations to clinical outcomes as in adults**,** indicating its likely value as a surrogate marker both for adult and pediatric HF.

ClinicalTrials.gov Identifiers: NCT00005391 (May 26, 2000), NCT01873976 (June 10, 2013).

## Introduction

Heart failure (HF) is a major contributor to morbidity and mortality worldwide. In the United States, the number of patients living with HF is expected to increase by nearly 50% between 2012 and 2030, at which time more than 8 million adults will be affected [1]. Despite substantial improvements in the medical and surgical treatment of HF over the past decades, mortality from HF remains high, with about 30% of adult HF patients dying within 1 year of hospitalization for the condition [2].

In children, HF is a common sequela of cardiomyopathies and congenital cardiovascular malformations. As in adults, morbidity and mortality are high in children with underlying cardiomyopathies and congenital cardiovascular malformations [3–7]. Treatments for children are largely extrapolated from adult HF guidelines [8, 9]; however, in randomized controlled trials of children, no medical therapies have improved survival or reduced the incidence of HF [10–12]. This circumstance, in part, reflects the challenges of drug trials in rare and vulnerable populations. Recruiting a sufficient sample size is often impractical, if not impossible, given the relatively low prevalence of HF in childhood and the years of follow-up needed to detect meaningful survival differences [11]. The availability of surrogate endpoints for mortality and morbidity in children with HF could greatly shorten the needed follow-up period and reduce the sample size, improving feasibility and the probability of a conclusive trial.

Natriuretic peptides are established diagnostic biomarkers for HF in children and adults [8, 9, 13]. Plasma N-terminal pro hormone B-type natriuretic peptide (NT-proBNP) concentrations are generally related to typical clinical endpoints, such as death and hospitalization [13–18], which allows drug-related reductions of NT-proBNP to predict the clinical effects of HF medications, independent of the cause of HF and other factors [19, 20]. Therefore, we sought to determine whether NT-proBNP concentrations could predict transplant-free survival in children with HF by comparing their data to recently published data for adults with HF [19].

## Methods

### Datasets

Data for this study came from two sources. Electronic medical record data for children with HF were extracted from the IBM Explorys™ database, which had also provided the data used in another study to evaluate adults with HF [19]. The Pediatric Cardiomyopathy Registry (PCMR) [NCT00005391] [21] provided registry data from the National Institutes of Health-supported PCMR sub-study called Pediatric Cardiomyopathy Biomarkers [NCT01873976] [22].

The period covered by Explorys is about 20 years; that is, records go back to about 2000. Data were abstracted on the 10th and 18th of March 2020 from a subsection of the database specifically comprising all records of patients with HF, as defined by the Systematized Nomenclature of Medicine, Clinical Terms (SNOMED-CT) concept ID 84114007.

Data for children meeting the following criteria were abstracted:

- Age at HF diagnosis less than 18 years
- At least one measurement of the plasma NT-proBNP concentration
- A specific SNOMED-CT-coded HF diagnosis
- Year of birth

The study endpoint was the composite of “all-cause death” or “heart transplant” (SNOMED codes 32413006, 47058000, or 32477003) over 2 years as in the adult cohort [19]. We wanted to study children with chronic HF at entry into the study. Accordingly, the baseline was set at 1 month after the first recorded HF diagnosis because the first diagnosis for HF is often associated with an acute event, such as decompensated HF. Mortality in such a crisis is higher in this comparatively short period, in which children often either recover or die [5, 18]. This definition of baseline means that the date of the first documented HF diagnosis is one month before the date of “enrollment” into our virtual real world data-based study. For simplicity, we will therefore refer to “enrollment date” for the remainder of this paper for PCMR and for Explorys. We used the median NP-proBNP value in the follow-up period for modeling.

The Pediatric Cardiomyopathy Biomarker sub-study was a prospective cohort study of children with dilated cardiomyopathy (DCM) [22]. Children were enrolled between 2013 and 2016 and followed for up to 5 years. The date of diagnosis was the date of the earliest diagnostic echocardiogram, cardiac magnetic resonance imaging study, or report that confirmed the diagnosis of DCM. Only children with a diagnosis of DCM at baseline were included in these analyses. As in the case of the Explorys cohort, the primary endpoint for children in the PCMR was the composite of all-cause death or heart transplantation, but the rates of events were determined within 5 years after enrollment in the registry to include a sufficient number of events because the incidence was lower than that in the Explorys population. The NT-proBNP values used for modeling were measured at the time of enrollment. Each participating center obtained Institutional Review Board or Ethics Committee approval for the study.

IBM Explorys is a commercial database fully compliant with the Health Insurance Portability and Accountability Act and the Health Information Technology for Economic and Clinical Health Act. Therefore, no approval by the institutions’ human research committee was required, and informed consent by the patients was not obtained. In the PCMR database, written informed consent was obtained from parents or guardians when children were enrolled.

### Statistical Modeling

In adults with HF irrespective of left ventricular ejection fraction (LVEF), an E_max_-type function describes the relationship between individual NT-proBNP concentrations and probabilities for clinical events [19]. The same equation was used to model the data for children because a comparable relationship was expected.

1 $${P}_{i}= {P}_{0}+{P}_{max} \times \frac{{{\text{NT}}-{\text{proBNP}}}_{{\text{i}}}^{\gamma }}{{{\text{NT}}-{\text{proBNP}}}_{{\text{i}}}^{\gamma }+{{{\text{EC}}}_{50}}^{\gamma }}$$

Where, *P*_i_: probability for an event, *P*_0_: baseline probability for an event when the NT-proBNP concentration is very low, *P*_max_: maximum probability added onto the baseline value, EC_50_: NT-proBNP concentrations corresponding to the half-maximum risk for events, *γ*: Shape parameter determining the steepness of the curve, NT-proBNP_i_: individual plasma NT-proBNP concentrations.

E_max_ functions are usually used to describe the concentration–response relationships of drugs and have a non-linear shape, flattening out at high concentrations and resulting in a maximal effect. In addition, the most general E_max_ function starts at a potential non-zero (placebo) effect at a concentration of zero and may have a more or less s-shape, as determined by the shape parameter *γ*. The location of the efficacy curve on the concentration axis is determined by the parameter EC_50_.

It is important to note that the parameters *P*_0_ and *P*_max_ cannot be expected to be independent of the scenario the observed data are derived from, while for EC_50_, this might very well be the case. Thus, both, *P*_0_ and *P*_max_, will in any case depend on the patients’ ages, as well as on their observation periods. Also co-morbidities may have an influence. However, the rise of the risk from baseline to the maximal risk with increasing NT-proBNP concentration, which is determined by EC_50_ and *γ*, may follow the same shape in different cases. Therefore, the model is not able to predict absolute risks, but within a patient cohort for which *P*_0_ and *P*_max_ are determined, the relative risks of different subgroups or individuals can be predicted.

Model parameters were estimated with a maximum likelihood estimator. The number of adjustable parameters was reduced if necessary for a stable regression. Confidence intervals were estimated using a local approximation of the variance–covariance matrix on finite difference-based second derivatives of the likelihood function. Confidence intervals for estimates were derived through forward propagation of the parameter uncertainty using 10,000 random samples drawn from the approximated variance–covariance matrix. All evaluations and modeling were done with the statistical analysis software R V 4.0.2 and SAS Enterprise Guide V8.3.

## Results

### Baseline Patient Characteristics

In the Explorys database, 109 records met the inclusion criteria and were included in the analysis. Of the 150 eligible children included in the PCMR, NT-proBNP data were available for 146 (Table 1).

**Table 1:** Baseline characteristics of children and adults with cardiomyopathy in the Explorys registry and of children in the PCMR

	Explorys child cohort, *n* = 109		PCMR cohort, *n* = 150	
Characteristic	*N*	Value	*N*	Value
Male *n* (%)	109	58 (53)	150	64 (43)
Age at enrollment*, median (IQR), years	109	3 (0, 14)	150	8 (2, 15)
Weight, median (IQR), kg	105	13 (6, 39)	148	25 (11, 58)
Height, median (IQR), cm	105	90 (60, 144)	148	127 (83, 163)
BMI, median (IQR), kg/m^2^	105	16.2 (14.6, 19.0)	148	18 (15, 22)
NYHA/Ross HF Class, *n* (%)			150	35 (23)
I		NA	35	19 (54)
II		NA	35	10 (29)
III		NA	35	2 (6)
IV		NA	35	4 (11)
Missing data, *n* (%)		109 (100)		115 (77)
Medication use, *n* (%)	109	109 (100)	150	150 (100)
ACE inhibitor		66 (61)		103 (69)
Beta-blocker		45 (41)		67 (45)
Antiarrhythmic		87 (80)		6 (4)
Diuretics		92 (84)		48 (32)
Glycoside		29 (27)		34 (23)
Inotropic agents		80 (73)		27 (18)
Angiotensin receptor blockers		2 (2)		4 (3)
eGFR, median (IQR), mL/min/1.73m^2^	73	108 (81, 127)	17	112 (70, 136)
LVEF%, median (IQR)	7	56 (21, 61)	78	29 (20, 40)
NT-proBNP, median (IQR), pg/ml	109	1250 (328, 3700)	146	184 (65, 1970)
Heart disease diagnoses, *n* (%)	109	109 (100)	150	150 (100)
Dilated cardiomyopathy, %				150 (100)
Congenital cardiovascular malformations		53 (49)		
Cardiomyopathy		39 (36)		
Clinical events				
Observation period, year		2		5
Death, *n* (%)	109	8 (7)	150	4 (3)
Transplantation, *n* (%)	109	24 (22)	150	24 (16)
HF Hospitalization, *n* (%)		NA	150	11 (7)

*In the case of Explorys the “age at enrollment” is the age one month after the first documented HF diagnosis

*ACE* angiotensin-converting enzyme, *BMI* body mass index, *eGFR* estimated glomerular filtration rate, *HF* heart failure, *LVEF* left ventricular ejection fraction, *NA* not available

By definition, children in the PCMR cohort all had DCM, whereas 36% of the children in Explorys cohort had cardiomyopathy and a larger group had congenital cardiovascular malformations. For example, 40% had congenital cardiovascular malformations. Median plasma NT-proBNP concentration was considerably higher in the Explorys cohort than in the PCMR cohort (1250 pg/mL vs. 184 pg/mL). The use of diuretics and inotropic agents was much higher in the Explorys cohort than in the PCMR cohort (85% vs. 32% and 73% vs. 18%, respectively). The percentages of children treated with other cardiovascular medications were similar in both cohorts. The Explorys cohort seemed to include more severely ill children, given the proportion receiving inotropic agents (73%) and their higher rates of death or heart transplantation.

**Fig. 1:**
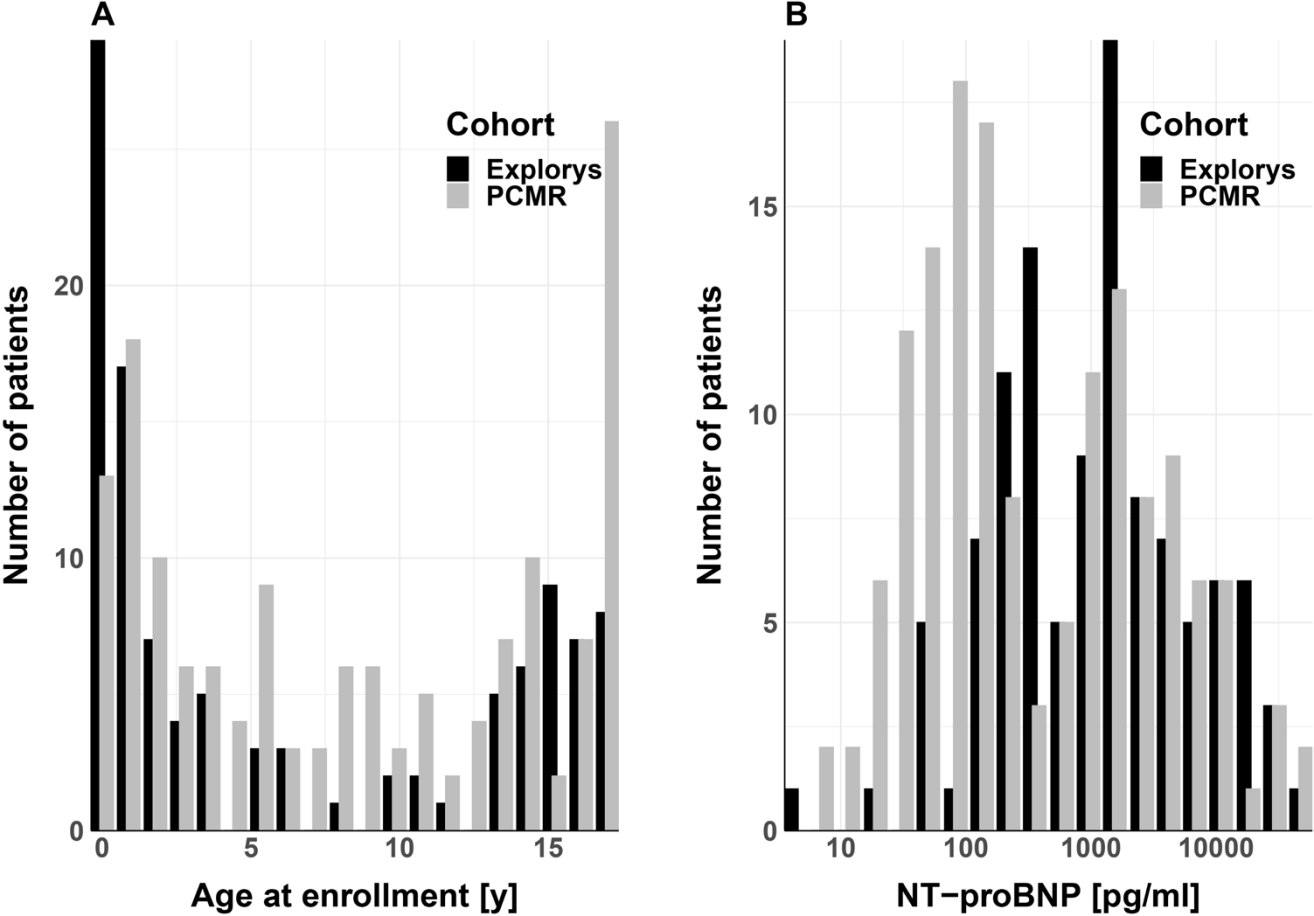
Comparison of age at enrollment and NT-proBNP distributions in both study cohorts

The age distributions were largely similar in the cohorts (Fig. 1). There was a bimodal age distribution in both cohorts. Interestingly, NT-proBNP concentrations were bimodal for both cohorts, in contrast to those of adults with HF, where the concentrations usually had a log-normal distribution [23]. The PCMR cohort includes more children with NT-proBNP concentrations below 400 pg/mL than does the Explorys cohort. The distribution of NT-proBNP concentrations above 400 pg/mL, however, is similar in both cohorts.

### NT-proBNP Concentrations and the Risk of Death or Transplant

**Fig. 2:**
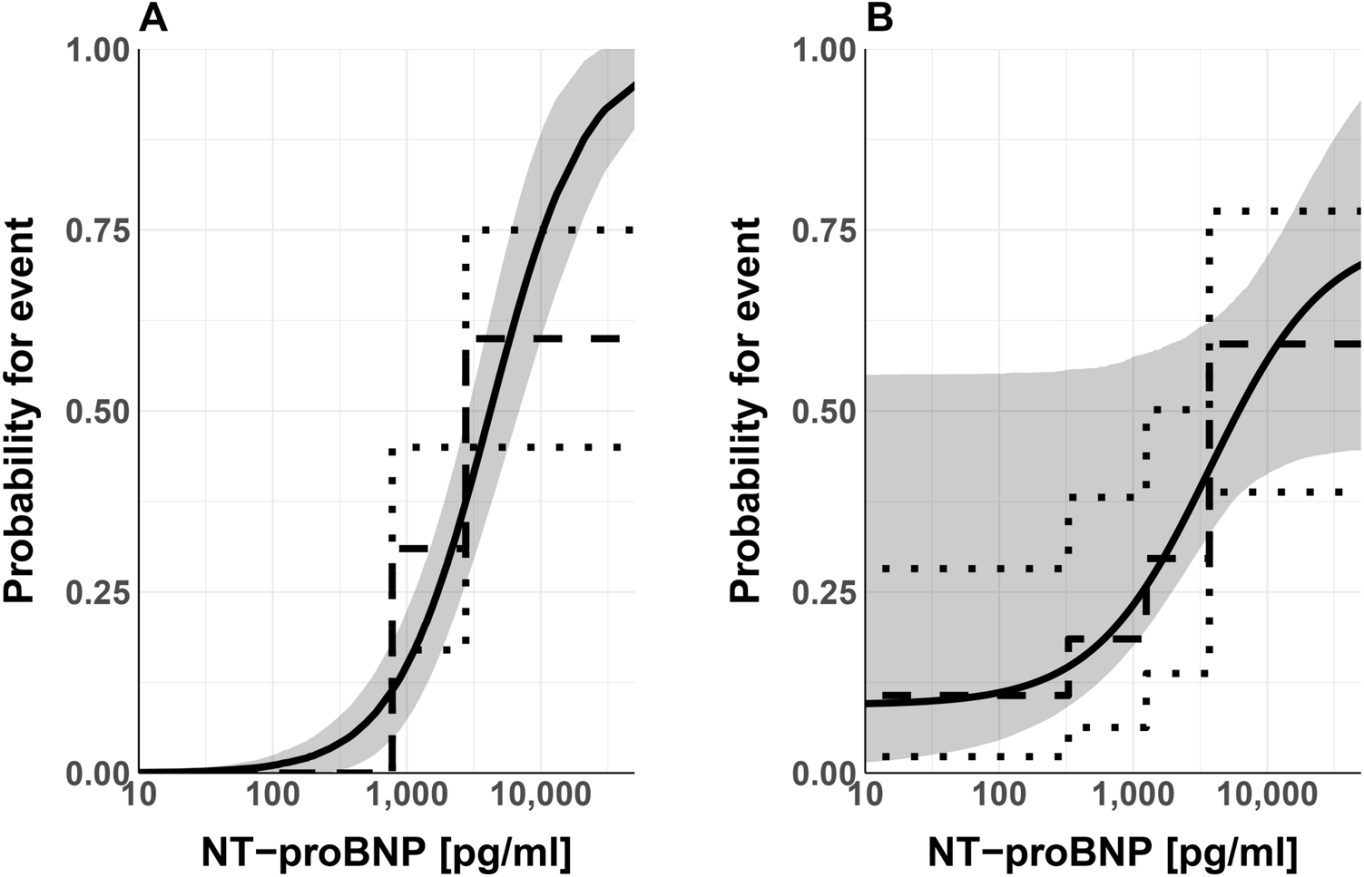
The dashed lines show the probabilities for occurrence of the clinical endpoint heart transplantation or death for the PCMR (**A**) and Explorys (**B**) cohorts in dependence of serum NT-proBNP concentrations. The steps represent the mean probabilities in quintiles (**A**) or quartiles (**B**) of the NT-proBNP concentrations in the respective cohorts. (There are no events in the lowest three quintiles of the PCMR cohort). The solid lines show *E*_max_-like model functions fitted to the individual event and NT-proBNP data. Dotted lines and gray areas represent the 90% confidence intervals of observed data and model predictions, respectively

The dashed and dotted lines in Fig. 2 represent event rates and 90% confidence intervals in NT-proBNP bins defined by quintiles (PCMR; 146 patients) and quartiles (Explorys; 109 patients) of the respective NT-proBNP distributions. Both curves feature a steep increase in event rates around NT-proBNP above ~ 1000 pg/ml. Note that in both cases and in particular in the case of the PCMR cohort there are little data in the high NT-proBNP range as it would be required for identifying the *P*_max_ value (1) with high accuracy.

The Explorys data could be described very well using the model described in Eq. 1 keeping the shape parameter *γ* fixed at 1 to keep the number of free parameters as small as possible. The estimated values of the optimized model parameters (90% CI) are *P*_0_ = 0.094 (0.065), EC_50_ = 3730 (3140) pg/mL, and *P*_max_ = 0.65 (0.18). The substantial width of the confidence intervals reflects the small number of patients and consequently, the large uncertainty in the data.

Simulated values for the PCMR cohort (Eq. 1) were also similar to the observed values (Fig. 2). In this cohort, however, the shape parameter *γ* was included in the parameter estimation, whereas *P*_0_ and *P*_max_ were fixed at 0 and 1, respectively. By doing this, the approach is equivalent to a log-logistic regression. The choice of *P*_0_ = 0 was justified because there was no event in children in the lowest quintiles of the NT-proBNP distribution. *E*_max_ on the other hand could not be identified in the optimization process because of a lack of data at very high NT-proBNP concentrations. The estimated values of the two remaining parameters in the model were 4199 pg/mL for EC_50_ and a mean (SE) of 1.21 (0.24) for *γ*.

### Comparing NT-proBNP-Derived Prognoses in Children to Those in Adults

The relationship between NT-proBNP concentration and mortality in adults with HF in the Explorys database [19] was determined with the same model equation used above, so the resulting models can easily be compared (Table 2).

**Table 2:** Estimates and respective standard errors (in parenthesis) for *E*_max_ model parameters (Eq. 1) for the relationship between plasma NT-proBNP concentrations and the probability of the endpoint (all-cause death or heart transplantation) in the two cohorts of children and adults with heart failure

Parameter	Pediatric model Explorys (*n* = 109)	Pediatric model PCMR (*n* = 146)	Adult model Explorys (*n* = 108,330)
*P*_0_	0.094 (0.065)	0^a^	0.055 (0.002)
EC_50_, pg/mL	3730 (3140)	4200 (1110)	3880 (149)
*P*_max_	0.65 (0.18)	1^a^	0.55 (0.01)
*γ*	1^a^	1.21 (0.24)	1^a^

^a^Values fixed in model optimization for reasons discussed in the text

*EC*_*50*_ NT-proBNP concentration relating to half-maximum risk for events*,* *γ* shape parameter determining steepness of curve*,* *P*_0_ baseline probability for event*,* *P*_max_ maximum added probability

**Fig. 3:**
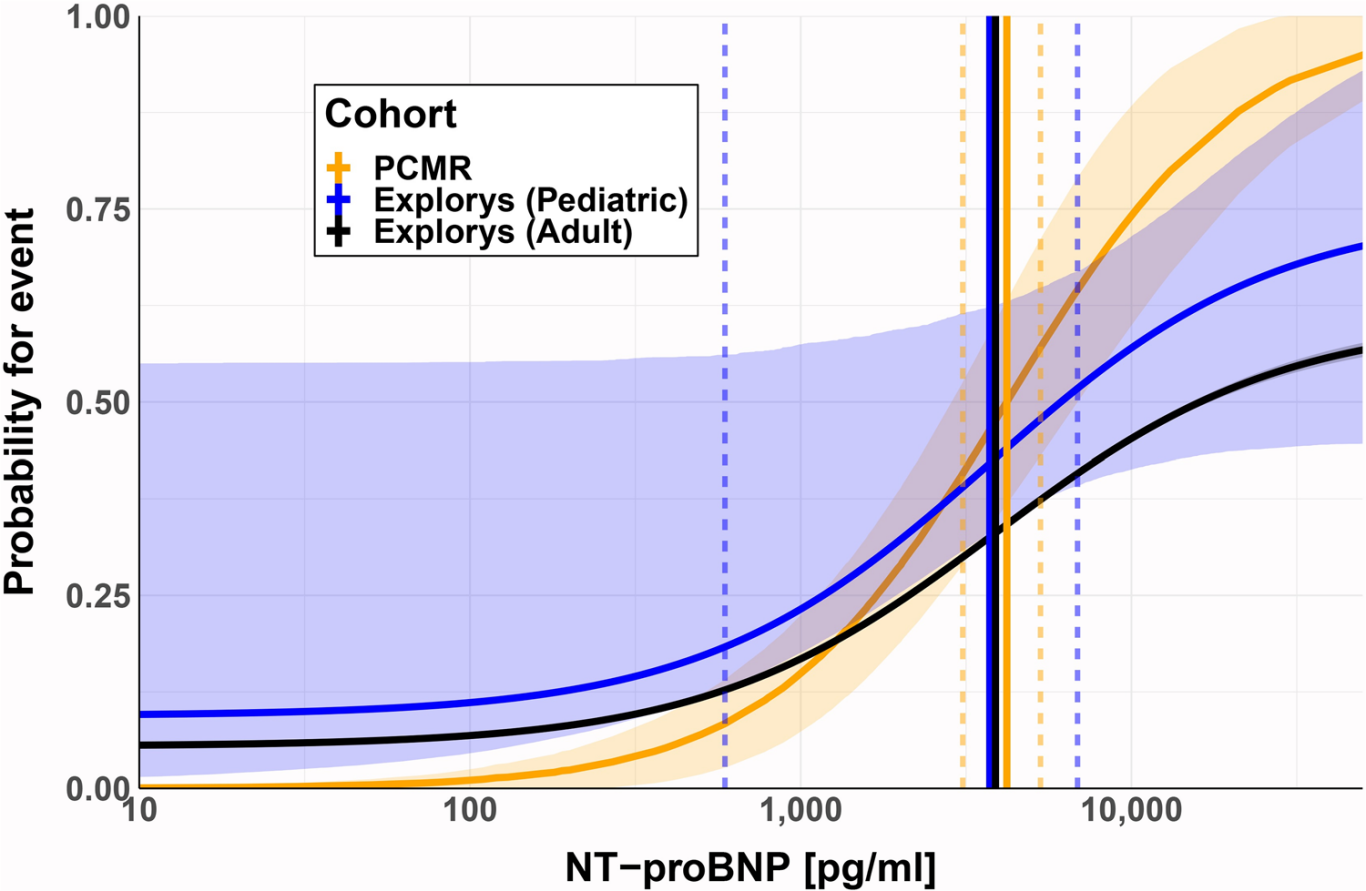
Comparison of probabilities of clinical events in pediatric and adult populations. The curves show model predictions of the probability for children (PCMR: orange, Explorys: blue) and adults (black) with heart failure reaching the composite endpoint of death or heart transplantation as predicted from NT-proBNP concentrations. The results for children are the same as in Fig. 2. Results for adults were calculated with the previously published model based on the Explorys data for adult heart failure patients [19]. Lines represent medians and 90% confidence intervals. The gray confidence intervals for adults is smaller than the width of the black line, given the large number of data included in the analysis. EC_50_ parameter estimates and respective standard errors are indicated as solid and dashed lines. Standard errors for the Explorys adult cohort are very small and have been omitted for clarity

In the Explorys database, the number of heart transplants in adults was negligible compared to deaths, so this endpoint was ignored in the comparison. None of the estimated parameters differed markedly between children and adults in the Explorys cohorts, as shown by their overlapping confidence intervals. This implies that the relationship between NT-proBNP and mortality and heart transplant is comparable in both cohorts (Fig. 3). In the case of the PCMR cohort, however, the fact that baseline and maximum probability for an event was fixed to 0 and 1 does not allow a statistical assessment of the deviation of these parameters from those for the other two cohorts. The EC_50_ values, however, are statistically indistinguishable in all three cases.

Respectively, all model curves (of both the pediatric and adult cohorts) coincide in the range of their EC_50_ values and their confidence intervals (Fig. 3). Differences in *P*_0_ between the adult and pediatric Explorys cohorts could be explained by the severity of the disease status of the affected children. The value of *P*_max_ for the PCMR cohort is not informed by the data, which was insufficient in the high NT-proBNP range and was thus set to 1 in the model. Therefore, the apparent deviation from the other two curves may be purely artificial and cannot be interpreted.

## Discussion

This large observational study of NT-proBNP concentrations in children with HF has several findings of interest. The PCMR cohort consisted exclusively of children with DCM, who tended to be younger and have less severe disease, as evidenced by the less frequent use of diuretics and inotropes, than those in the Explorys cohort. Conversely, the Explorys cohort included pediatric patients with diverse underlying cardiac diseases leading to HF, including nearly 50% with congenital cardiovascular malformations. Still, the association of NT-proBNP concentrations with the primary endpoint of death or heart transplant was similar across NT-proBNP concentrations, indicating that the risk of a cardiac event might be less determined by the type of cardiac disease than by myocardial stretch and stress, as measured by the NT-proBNP concentration. Cases with normal NT-proBNP concentrations (less than 100 pg/mL) had less than a 25% probability of reaching the primary endpoint over the 2- or 5-year follow-up period, whereas those with concentrations greater than 10,000 pg/mL had a 50% to 75% probability of reaching the primary endpoint.

We appreciate that these conclusions are semi-quantitative given that the datasets are too small for a rigorous model based comparison of the two cohorts as reflected by the large parameter uncertainties and the fact that we could not identify P_max_ in the PCMR model. On the other hand in light of the unavailability of larger datasets in pediatric heart failure, we are still adding substantial value by the findings that we could establish here.

Remarkably, our findings are similar to those of adults with HF with reduced or preserved LVEFs from the Explorys database [19]. Therefore, we conclude that the relationship between NT-proBNP concentrations and the risk for cardiac events relative to baseline risk is widely independent of age and HF etiology.

An inactive form of B-type natriuretic peptide is released from cardiomyocytes in response to myocardial stretch [24]. Myocardial stretch may be the result of abnormal left ventricular loading conditions and, when elevated increases the likelihood of HF and of clinically important events, such as heart transplantation or death. This finding is consistent with those of the few studies of children with cardiomyopathy and congenital cardiovascular malformations [14, 25–27].

The baseline characteristics of the two cohorts we investigated reflected a typical age distribution comprising mainly infants and teenagers, with a smaller number of children 4 to 12 years old. A recent analysis of the US Pediatric Health Information System database identified 67,349 children up to 19 years old with HF, of which 87% had chronic heart disease and 6% had cardiomyopathy. Of the total, 75% and 21% of the infants less than 1 year old and 8% and 49% of the teenagers (10 to 17 years old) had congenital cardiovascular malformations and cardiomyopathy, respectively [28]. Infants (47%) and young children (1 to 5 years old; 23%) were also overrepresented in a recent multicenter cohort study from the Netherlands on the outcomes of children with DCM [15]. In a post hoc analysis of a large pediatric carvedilol trial, 40% of patients were younger than 2 years of age, whereas the number of those between 2 and 18 years old were almost equally distributed [12, 15]. The distribution of NT-proBNP concentrations in these children was, however, almost log normal, with a very low mean of 90 pg/mL, in contrast to the bimodal distributions we found in our two cohorts.

In a recent analysis of 910 patients with congenital cardiovascular malformations from one tertiary center [20], 59% were younger than 1 year of age, whereas 18% were 1–10 years of age and 23% 10–18 years of age. 138 patients experienced a major adverse cardiovascular event (MACE) event (defined as death, resuscitation, mechanical circulatory support, or hospitalization caused by cardiac decompensation) with most events happening within the first 2 years after baseline (the earliest point with full set of laboratory data and medical records available). Patients experiencing a MACE had a median NT-proBNP of 10,950 pg/ml (IQR 4723–26,825) in contrast to patients not experiencing a MACE, who had a median NT-proBNP of 1,130 pg/ml (IQR 133–6073).

In children with DCM or complex congenital cardiovascular malformations, the risk for dying or receiving a heart transplant is closely related to NT-proBNP concentrations. Moreover, as demonstrated here, the relationship between the NT-proBNP concentration and clinical events in both cohorts can be described by a mathematical model derived from adults with HF, although these small datasets still leave uncertainty as demonstrated by the large standard errors as well as the difficulties to identify *P*_max_ in the PCMR cohort. On the other hand, the good agreement between simulated and observed values, and the fact that the risk for a clinical endpoint for similar NT-proBNP concentrations was similar in both cohorts, supports the validity of the model assumptions and in conclusion suggests that the relationship we found is independent of the underlying disease. Importantly, the relationship we found for children is similar to that determined for adults with diverse HF etiologies, in whom the relationship could be predicted with much higher confidence.

This analysis confirms the hypothesis derived from single-center reports pointing to similar risk categories defined by NT-proBNP thresholds [16, 29–31] and their association between lower NT-proBNP concentrations and improved outcomes [16, 18].

### Limitations

We combined data from two small cohorts of children with HF from different causes. Whereas the Pediatric Cardiomyopathy Biomarker sub-study of the PCMR was prospective and had pre-specified sampling timepoints, the Explorys analysis was retrospective and used NT-proBNP concentrations as collected. Furthermore, the PCMR cohort met pre-specified enrollment criteria, whereas the Explorys cohort was selected only by diagnostic codes resulting in a less well-defined group. Heart failure in children in both cohorts could be new or chronic. For children in the Explorys cohort, we used the median concentration of the available NT-proBNP values in the follow-up period, whereas we used the NT-proBNP concentration at enrollment for children in the PCMR cohort. Additionally, serial data were not available. Such data are needed to assess the predictive value of changes in NT-proBNP concentrations and whether natriuretic peptides or other biomarkers could be part of goal-directed therapy [32]. Furthermore, as described in the discussion both datasets were too small for testing equivalence of models in a statistically robust sense and hence, the assessment has to be semi-quantitative.

A possible limitation is that there is a substantial number of pediatric heart failure patients with congenital cardiovascular malformation diagnoses in the Explorys database. This may be viewed as somewhat concerning as the cause for serum NT-proBNP concentrations theoretically could be increased due to this (such as shunting) beyond what might be seen in DCM (pump failure). We have previously compared the association between serum NT-proBNP concentrations in children with heart failure from with DCM with children with heart failure with congenital cardiovascular malformations and published that when we compared the entire sample to the subgroup of children with DCM without congenital cardiovascular malformations assess confounding we found that NT-proBNP increased significantly with the severity of symptoms with a volume under the receiver operating characteristic surface estimate of 58% which was significantly more frequent than what would have been expected for an uninformed marker [31, 33]. However, we did find that when patients with congenital cardiovascular malformations were excluded NT-proBNP still increased significantly with the severity of symptoms but its discriminatory ability was lower [33]. We concluded that comparing the effect estimates in the entire sample to those within the subgroup of children with congenital cardiovascular malformations may have confounded the associations between NT-proBNP and symptom severity in the direction of minimizing a statistically significant association [33]. Therefore, theoretically comparing the Explorys cohort with thee PCMR cohort may have a bias toward not having a significant association. Since these two groups of pediatric HF patients were quite similar in this analysis, this suggests that the true association of serum NT-proBNP concentration in pediatric HF patients from either DCM or congenital cardiovascular malformations may be similar and in fact may be even more significant in pediatric DCM HF patients that demonstrated here since the direction of relation to symptoms in congenital cardiovascular malformation patients may be lower. In our prior work, we found that there were differences between children with HF from DCM when compared to children with HF from congenital cardiovascular malformations in terms of medications and measurements of LV structure and function [31, 33]. Nevertheless, these groups were comparable using serum NT-proBNP concentrations with respect to HF severity in our prior analyses [33] and in terms of mortality in this paper. So, although this is a limitation the serum NT-proBNP concentrations between these two pediatric HF populations and an adult HF population are important comparisons.

## Conclusion

Our findings may have important implications for developing new treatments for children with HF, especially because new therapeutic approaches are most often established in adults before being investigated in children. Therefore, by the time clinical trials commence in children, most often the drug’s safety, efficacy, and the value of NT-proBNP as the primary biomarker are thoroughly understood in adults. We found that NT-proBNP concentrations are significantly associated with the relative risks for mortality and HF in children are similar to those in adults and thus can help extend treatments from adults to children [34–36].
